# Effects of Drought on Nutrient Uptake and the Levels of Nutrient-Uptake Proteins in Roots of Drought-Sensitive and -Tolerant Grasses

**DOI:** 10.3390/plants7020028

**Published:** 2018-03-30

**Authors:** Deepesh R. Bista, Scott A. Heckathorn, Dileepa M. Jayawardena, Sasmita Mishra, Jennifer K. Boldt

**Affiliations:** 1 Department of Environmental Sciences, University of Toledo, Toledo, OH 43606, USA; deepesh.bista@rockets.utoledo.edu (D.R.B.); Dileepa.Jayawardena@rockets.utoledo.edu (D.M.J.); 2 Department of Biology, Kean University, Union, NJ 07083, USA; samishra@kean.edu; 3 United States Department of Agriculture, Toledo, OH 43606, USA; Jennifer.boldt@ars.usda.gov

**Keywords:** climate change, drought, nutrient uptake, nutrient-uptake protein, roots

## Abstract

Climate change will increase drought in many regions of the world. Besides decreasing productivity, drought also decreases the concentration (%) of nitrogen (N) and phosphorous (P) in plants. We investigated if decreases in nutrient status during drought are correlated with decreases in levels of nutrient-uptake proteins in roots, which has not been quantified. Drought-sensitive (*Hordeum vulgare*, *Zea mays*) and -tolerant grasses (*Andropogon gerardii*) were harvested at mid and late drought, when we measured biomass, plant %N and P, root N- and P-uptake rates, and concentrations of major nutrient-uptake proteins in roots (NRT1 for NO_3_, AMT1 for NH_4_, and PHT1 for P). Drought reduced %N and P, indicating that it reduced nutrient acquisition more than growth. Decreases in P uptake with drought were correlated with decreases in both concentration and activity of P-uptake proteins, but decreases in N uptake were weakly correlated with levels of N-uptake proteins. Nutrient-uptake proteins per gram root decreased despite increases per gram total protein, because of the larger decreases in total protein per gram. Thus, drought-related decreases in nutrient concentration, especially %P, were likely caused, at least partly, by decreases in the concentration of root nutrient-uptake proteins in both drought-sensitive and -tolerant species.

## 1. Introduction

Human activities are increasing the concentration of greenhouse gases in the atmosphere, causing the mean surface temperature of the Earth to warm, with the mean temperature expected to rise by 1.4–5.8 °C by the end of this century [1,2]. Though increasing temperatures will increase global precipitation, many regions of the Earth will experience decreases in precipitation and increases in the frequency, intensity, and duration of drought [1,2,3,4]. Increases in drought during the growing season, driven by reductions in precipitation and/or increases in evaporation, will decrease crop production at a time when human population is growing [1,2,3,4,5,6].

Increases in drought with global climate change will decrease plant growth, thereby decreasing food production in both natural ecosystems and agricultural systems. As plants are the main source of food for most humans [7], increases in drought will increase human hunger, and this will be exacerbated by population growth [6]. In addition, drought, heat stress, and high CO_2_ all tend to decrease the concentration of most nutrients in plant tissue (including in seeds) [8,9,10,11]. World-wide, more than two billion people already suffer from iron and zinc deficiency [7,12], since most plant tissue has low concentrations of these and many other nutrients, including the cereal grains that provide most of the calories for humanity [7]. Hence, the proportion of humanity suffering from malnutrition, which is caused by insufficient quantity or quality of food (especially protein, vitamins, and mineral nutrients) [11], is likely to increase in the coming decades due to climate change.

For plants, drought is one of the most common environmental stresses, and it negatively impacts their growth, development, and reproduction [4,13,14]. The impact of a drought depends on its intensity and duration, as well as on when it occurs during a plant’s life cycle [13]. A decrease in growth is the most-obvious plant response to water stress, which results from decreases in water uptake by roots, leading to a decrease in leaf expansion and reduced stomatal conductance, causing a decrease in C assimilation via photosynthesis [13,15]. In general, drought during the flowering and grain-filling stages has the most marked effects in terms of yield loss [14,16,17]. Drought can also have a strong impact on plant nutrient relations. For example, a recent meta-analysis [10] demonstrated that drought stress decreases the concentration of nitrogen (N) and phosphorus (P) in plant tissue, and several studies have shown that drought can decrease nutrient uptake from soil [18,19,20,21]. Decreases in nutrient uptake during drought may occur for several reasons, including the reduction of nutrient supply through mineralization [22,23,24], and by reducing nutrient diffusion and mass flow in the soil [25,26]. Drought could also decrease nutrient uptake by affecting the kinetics of nutrient uptake by roots, but this has been little studied [13,15,27].

Most plants obtain most of their mineral nutrients from the soil and transport these nutrients, using nutrient-uptake proteins located in the cellular membranes of roots and shoots. For example, in most plants, most of the N is taken up as inorganic nitrate (NO_3_^−^) or ammonium (NH_4_^+^), with lesser amounts taken up as amino acids or urea [26,28]. Nitrate is taken up by members of the NRT1 (low-affinity NO_3_^−^ transporters, at high N levels) and NRT2 (high-affinity NO_3_^−^ transporters, at low N levels) families of transporters, and ammonium is taken by the high-affinity AMT1 family [28]. Most phosphorus (P) is taken up by roots via the activity of PHT1-type transport proteins [29]. Schroeder et al. (2013) [12] highlighted the potential to improve the quantity and quality of plant production by altering transport proteins in plants, like manipulating iron and zinc transporters to improve nutritional quality, sodium or potassium transporters to improve tolerance to salinity, and aluminum (Al) transporters to increase tolerance to Al toxicity. However, the effects of drought on the level and activity of nutrient-uptake proteins have been little studied.

To improve our basic understanding of plant responses to drought, we investigated the effects of drought on the concentration of nutrient-uptake proteins in roots and how this relates to effects on nutrient-uptake rate and tissue nutrient concentration, specifically focusing on N and P. We examined the impacts of drought in three grass species encompassing a range of drought tolerance (barley and corn, which are sensitive, compared to *Andropogon gerardii*, or big bluestem, which is drought tolerant), collecting data at both mid- and late-drought. Drought-sensitive and -tolerant species are likely to differ in their nutrient-relations responses to drought for several reasons [13,26], including the response of %N and P, due to a hypothesized balanced effect of drought on C gain vs. N and P uptake with longer drought, as would be experienced by drought-tolerant species, compared to greater negative effects on N and P uptake vs. C gain with short-term drought, as would be experienced by drought-sensitive species [10]. It is also possible that drought-tolerant species are able to maintain higher levels of protein synthesis, and hence nutrient-uptake proteins in roots, during drought than drought-sensitive species, though this has not been examined [10,13,15,26]. Given the knowledge gaps discussed above, we tested two main hypotheses: (a) Drought will decrease the concentration of nutrient-uptake proteins in roots, and this will correlate with decreases in nutrient-uptake rate; and (b) The effects of drought stress on nutrient uptake and nutrient-uptake proteins will differ in tolerant and susceptible plant species, with nutrient uptake in tolerant species being more resistant to drought.

## 2. Results

**Figure 1:**
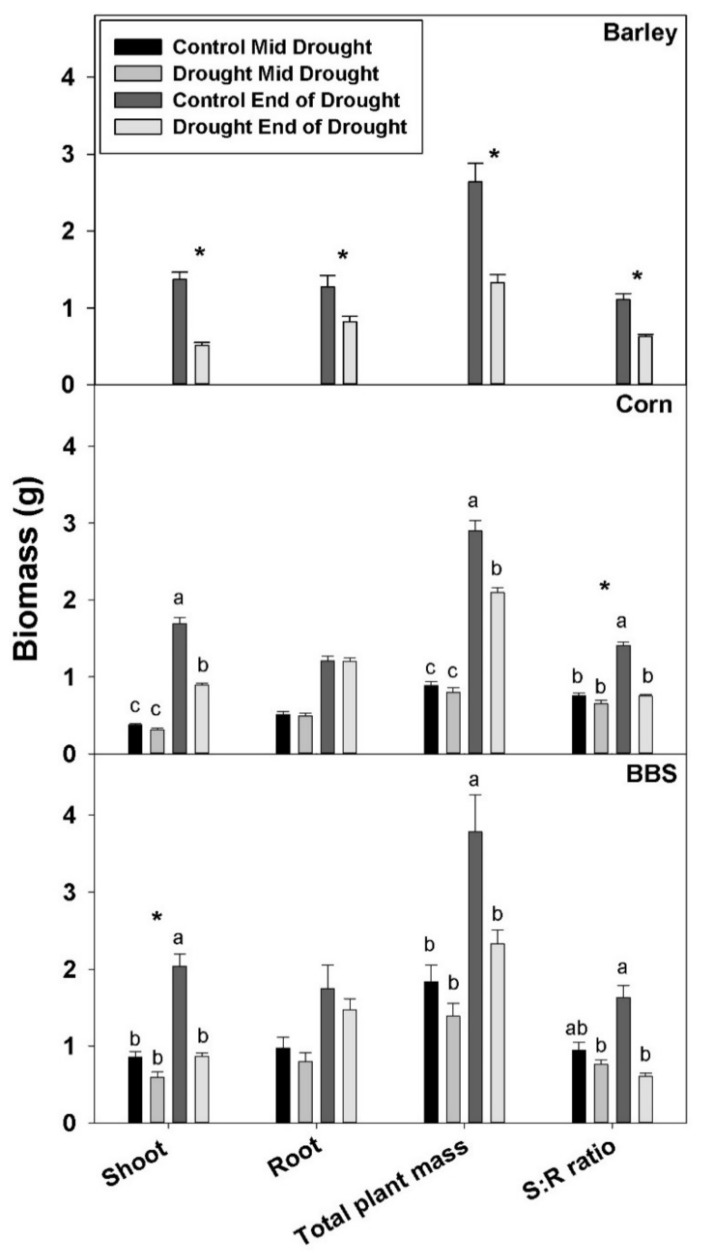
Effects of control vs. drought treatments on shoot, root, and total plant dry mass, and shoot:root (S:R) mass ratio in barley, corn, and big bluestem (BBS). Each bar represents mean + 1 SE (standard error). Within each panel, bars not sharing the same letters are significantly different. For each response variable, significant effects of drought (ANOVA), across both harvest in corn and big bluestem, are indicated with an asterisk (*). Note that only one harvest (end of drought) was conducted for barley because of its drought sensitivity.

By the “end-of-drought” harvest, the drought treatment had decreased shoot and total plant biomass in all three species, but it reduced root biomass only in barley; drought had no significant effect on biomass at the mid-drought harvest in corn and big bluestem (Figure 1, Tables S1–S3). The decrease in total plant mass at the end-of-drought harvest was 44% for barley, 28% for corn, and 38% for big bluestem, indicating that the relative severity of the drought was roughly comparable for each species (though, of course, the absolute severity of the stress, as measured by leaf water potential, was greater for big bluestem, as explained in the methods). As a consequence of large reductions in shoot mass, but smaller reductions in roots mass, drought decreased shoot-to-root biomass ratio in all three species by the “end-of-the-drought” (Figure 1, Tables S1–S3). The decrease in shoot-to-root mass was largest in big bluestem (62%), the most-drought-tolerant species, intermediate in corn (45%), and smallest in barley (39%).

**Figure 2:**
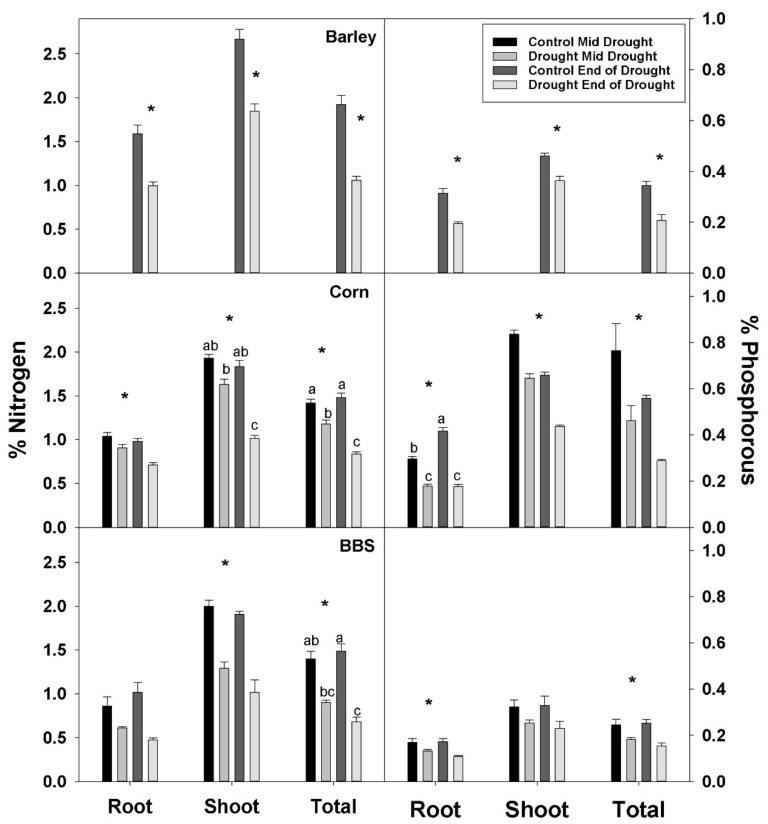
Effects of control vs. drought treatments on %N and %P per dry mass in root, shoot, and total plant tissue in barley, corn, and big bluestem (BBS). Each bar represents mean + 1 SE (standard error). Within each panel, bars not sharing the same letters are significantly different. Within each panel, bars not sharing the same letters are significantly different. For each response variable, significant effects of drought (ANOVA), across both harvest in corn and big bluestem, are indicated with an asterisk (*).

Shoot, root, and total plant %N were significantly decreased by drought in all three species, though the effects were sometimes not significant until the end-of-drought harvest in corn and big bluestem (Figure 2, Tables S1–S3). Similarly, drought decreased shoot, root, and total plant %P in all species, excluding for shoot %P in big bluestem (Figure 2, Tables S1–S3). Drought decreased total plant %N by 45, 44, and 51%, while %P decreased 41, 48, and 39%, in barley, corn, and big bluestem, respectively, by the end-of-drought; hence, drought effects on %N and %P were similar in magnitude in drought-sensitive and -tolerant species in this study. The magnitude of the decreases in %N and %P were similar for shoot, root, and whole plant, in all three species, indicating that decreases in shoot %N and %P were not attributable to effects of drought on translocation of nutrients from roots to shoot.

**Figure 3:**
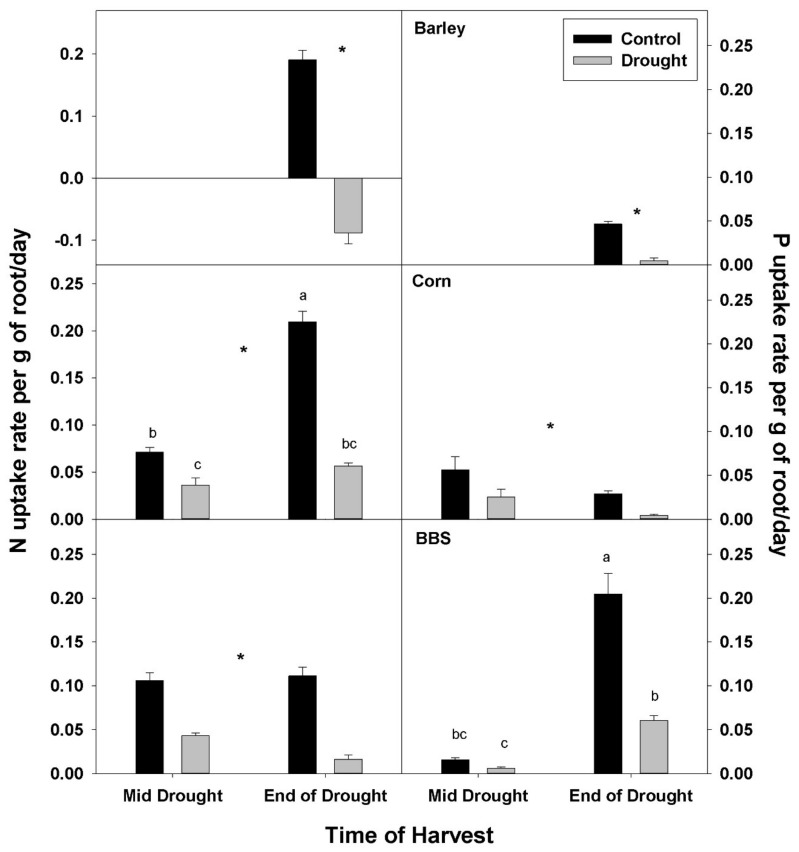
Effects of control vs. drought treatments on N and P uptake rate of roots in barley, corn, and big bluestem (BBS). Each bar represents mean + 1 SE (standard error). Within each panel, bars not sharing the same letters are significantly different. Within each panel, bars not sharing the same letters are significantly different. For each response variable, significant effects of drought (ANOVA), across both harvest in corn and big bluestem, are indicated with an asterisk (*).

The uptake rate of both N and P by roots was decreased by drought in all three species. Decreases in N- and P-uptake rate were larger for the end-of-drought, compared to the mid-drought, harvest, as might be expected with decreasing mobility of soil nutrients and plant water uptake as drought progressed (Figure 3, Tables S1–S3). In corn, N-uptake rate was decreased by drought 46% at mid-drought and 72% at end-of-drought, while P uptake was decreased 54% and 80% at the respective harvests. In big bluestem, N-uptake rate was decreased by drought 58% at mid-drought and 84% at end-of-drought, while P uptake was decreased 67% and 70% at the respective harvests. Decreases in N- and P-uptake rate with drought were larger in barley compared to corn and big bluestem, with N uptake decreasing 142% in barley, 72% in corn, and 84% in big bluestem at the end-of-drought harvest, while P uptake decreased 88%, 80%, and 70%, in barley, corn, and big bluestem, respectively. N-uptake rate in barley at the end-of-drought harvest was negative, indicating that barley lost N during drought.

**Figure 4:**
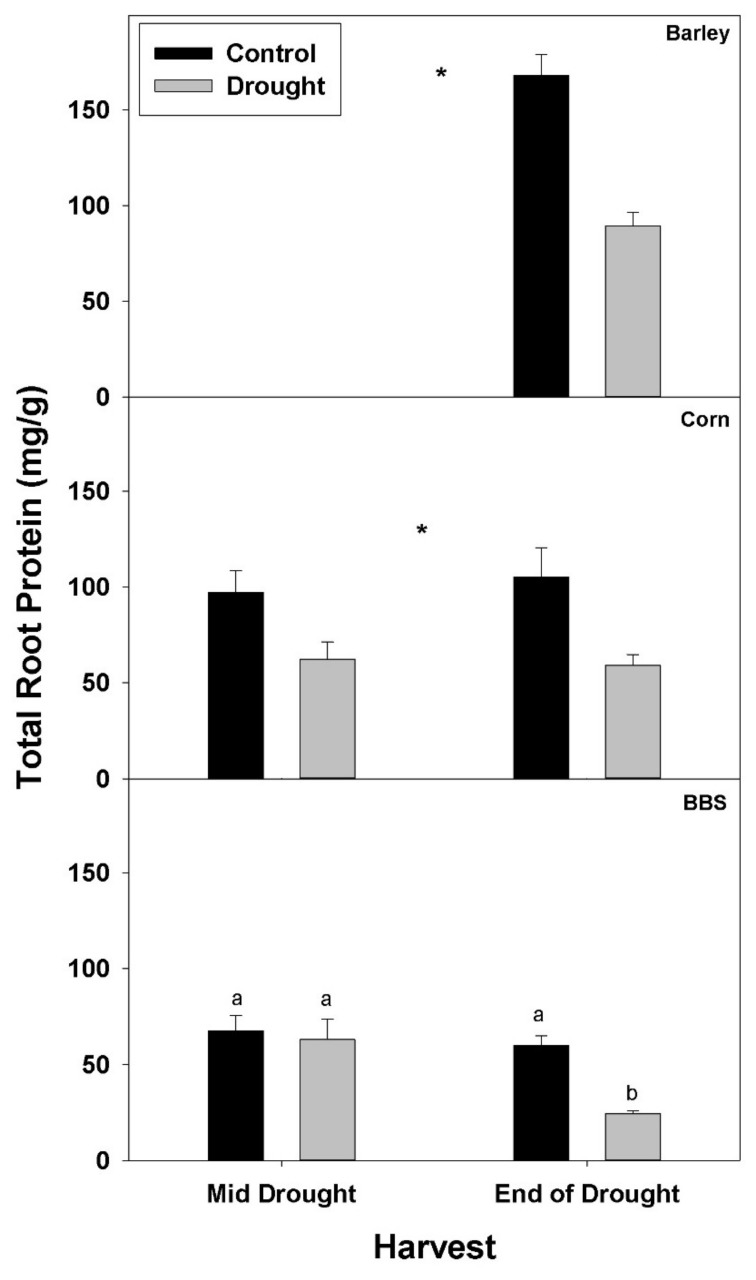
Effects of control vs. drought treatments on the concentration of total protein per dry mass of roots in barley, corn, and big bluestem (BBS). Each bar represents mean + 1 SE (standard error). Within each panel, bars not sharing the same letters are significantly different. For each response variable, significant effects of drought (ANOVA), across both harvest in corn and big bluestem, are indicated with an asterisk (*).

Drought significantly decreased the concentration of total protein in roots of all three species by the end-of-drought harvest (Figure 4, Tables S1–S3). As with %N and %P and N- and P-uptake rates, decreases in total root protein concentration with drought were larger at the end-of-drought than at mid-drought, (35 vs. 43% in corn and 6 vs. 60% in big bluestem, for mid- vs. end-of-drought, respectively). The decrease in total root protein concentration with drought was largest in big bluestem, declining 60% at the end-of-drought harvest, compared to 43% for barley and 47% for corn.

**Figure 5:**
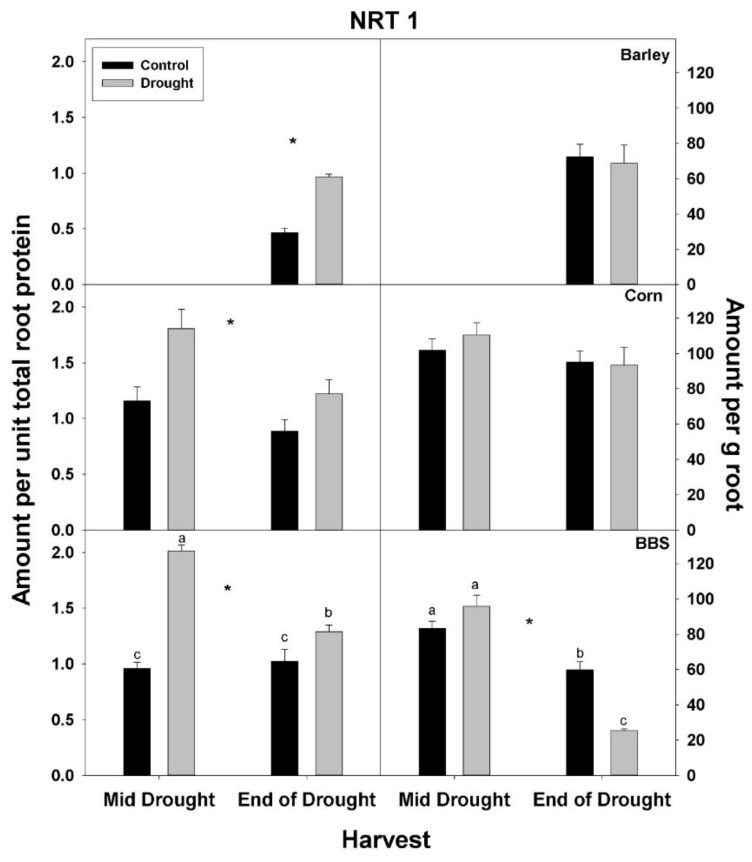
Effects of control vs. drought treatments on the concentration of the nitrate-uptake protein, NRT1, of roots in barley, corn, and big bluestem (BBS). Each bar represents mean + 1 SE (standard error). Within each panel, bars not sharing the same letters are significantly different. Within each panel, bars not sharing the same letters are significantly different. For each response variable, significant effects of drought (ANOVA), across both harvest in corn and big bluestem, are indicated with an asterisk (*).

**Figure 6:**
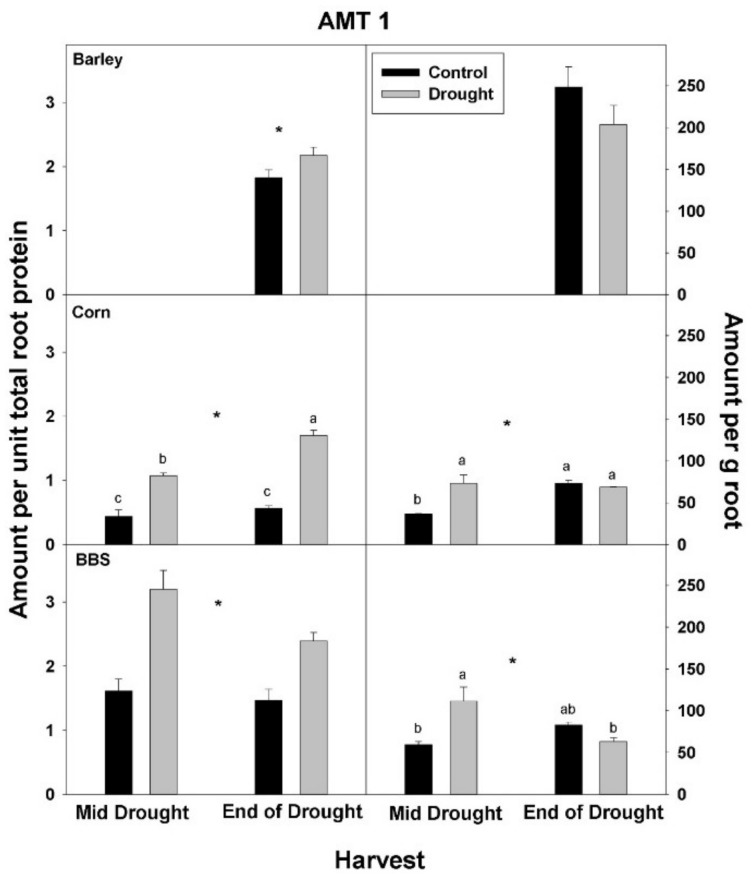
Effects of control vs. drought treatments on the concentration of the ammonium-uptake protein, AMT1, of roots in barley, corn, and big bluestem (BBS). Each bar represents mean + 1 SE (standard error). Within each panel, bars not sharing the same letters are significantly different. Within each panel, bars not sharing the same letters are significantly different. For each response variable, significant effects of drought (ANOVA), across both harvest in corn and big bluestem, are indicated with an asterisk (*).

**Figure 7:**
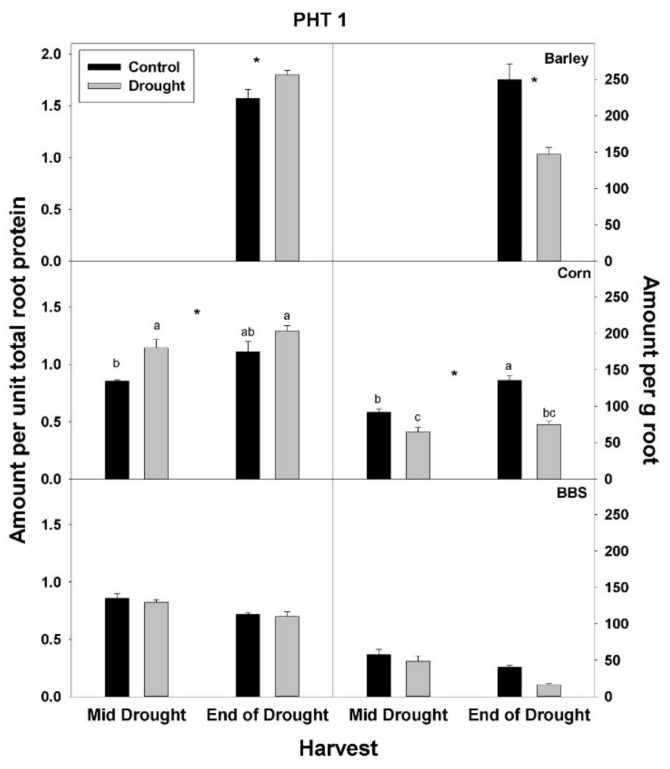
Effects of control vs. drought treatments on the concentration of the phosphate-uptake protein, PHT1, of roots in barley, corn, and big bluestem (BBS). Each bar represents mean + 1 SE (standard error). Within each panel, bars not sharing the same letters are significantly different. Within each panel, bars not sharing the same letters are significantly different. For each response variable, significant effects of drought (ANOVA), across both harvest in corn and big bluestem, are indicated with an asterisk (*).

**Figure 8:**
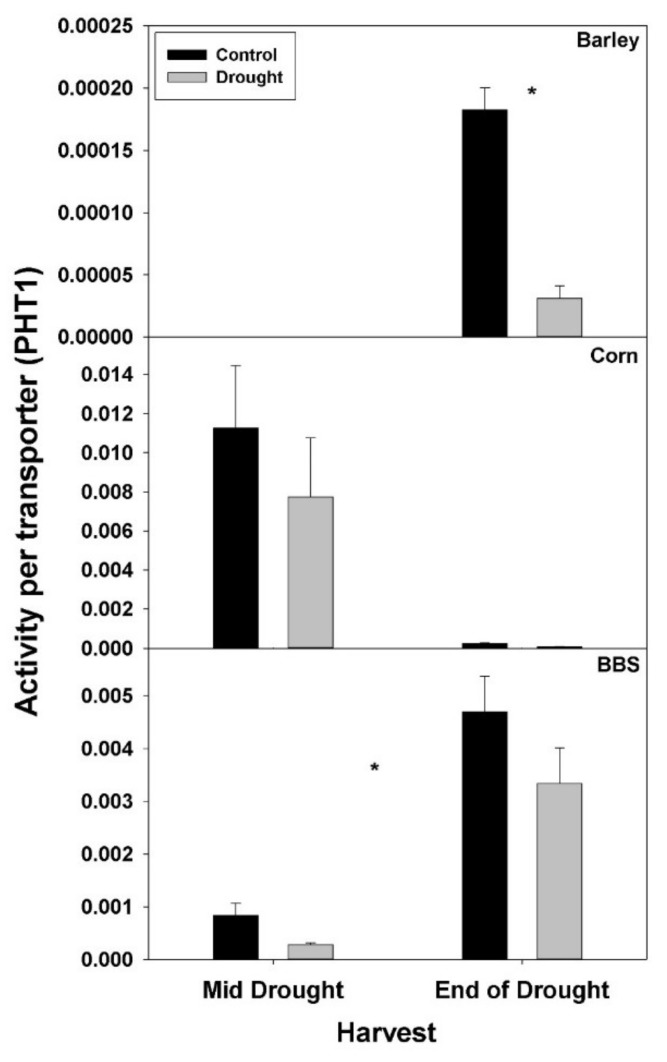
Effects of control vs. drought treatments on the relative activity of the phosphate-uptake protein, PHT1, of roots in barley, corn, and big bluestem (BBS). Each bar represents mean + 1 SE (standard error). Within each panel, bars not sharing the same letters are significantly different. Within each panel, bars not sharing the same letters are significantly different. For each response variable, significant effects of drought (ANOVA), across both harvest in corn and big bluestem, are indicated with an asterisk (*).

In contrast to total root protein, the concentration of NRT1, AMT1, and PHT1 per unit total root protein was significantly up-regulated (or marginally so, *p* < 0.10) with drought in all three species and at all harvests, excluding for PHT1 in big bluestem (Figure 5, Figure 6 and Figure 7, Tables S1–S3). No consistent effect of drought on nutrient-uptake protein per unit total protein was observed for either mid- vs. end-of-drought or across species. As a consequence of the increases in NRT1 and AMT1 per unit total root protein, the concentration of NRT1 and AMT1 per g root mass never decreased significantly with drought, excluding for NRT1 in big bluestem at the end-of-drought harvest (Figure 5 and Figure 6, Tables S1–S3). However, the concentration of PHT1 per g root mass decreased with drought in all three species, by the end-of-drought harvest, declining 40%, 44%, and 59% in barley, corn, and big bluestem, respectively (no consistent differences were evident between harvests). As with PHT1 per gram root, the relative activity of PHT1 decreased with drought in all species and at both harvests, with no consistent pattern evident for mid- vs. end-of-drought or among species (Figure 7 and Figure 8, Tables S1–S3).

## 3. Discussion

As a consequence of global warming caused by anthropogenic increases in greenhouse gases, many regions of the world are expected to experience a reduction in precipitation and an increase in evapotranspiration rates in coming decades, resulting in increased drought [1,2]. In addition to decreasing plant growth and reproduction, drought also decreases the concentration (%) of N and P in plant tissues [10], but the reasons for this decrease are not fully understood. In this study, it is demonstrated for the first time that drought decreases the concentration or activity of the major uptake-proteins for N and P (NRT1, AMT1, and PHT1), and these negative impacts on nutrient-uptake proteins provide a potential explanation for the observed reductions in N- and, especially, P-uptake rates by roots during drought, which can explain why plant %N and %P were decreased by drought. In addition, and as expected, while the effects of drought were similar for mid- vs. late-drought, in terms of pattern (increase/decrease/no change), the magnitude of effects were often greater late in the drought, when water stress was more severe, compared to earlier in the drought. Hence, the more severe the drought, the greater the negative impact on nutrient relations.

In this study, drought significantly reduced the concentration (%) of N by 44–51% and P by 39–48% by the end-of-drought harvest, in both drought-sensitive barley and corn and drought-tolerant big bluestem. Since drought slowed but did not prevent growth relative to well-watered controls, the reductions in whole-plant %N and %P with drought indicate that drought reduced the acquisition of nutrients more than it did the acquisition of water, and, hence, plant growth; consequently, decreases in nutrient acquisition cannot be explained simply by decreases in water uptake. Consistent with the observed decreases in %N and P with drought, the rate of N and P uptake per gram of root decreased with drought too, providing a potential explanation for the decreases in nutrient concentration. It has been recognized for decades that drought can decrease the rate of nutrient uptake by plants, independent of water uptake [13,15,30]. For example, drought might decrease water uptake in the upper soil layers, in which soil nutrient concentrations are often higher, before affecting water uptake from deeper soil layers [13]. Drought can also decrease soil nutrient concentrations by decreasing soil microbial activity [23,24]. In addition, drought can potentially decrease nutrient-uptake kinetics per unit root, such as by decreasing the activity of enzymes involved in nutrient assimilation, which might then slow nutrient uptake [15,31], or by decreasing expression of nutrient-uptake proteins in roots [30].

Consistent with the possibility that drought decreases the concentration or activity of nutrient-uptake proteins, drought decreased the concentration of the main P-uptake protein, PHT1, per gram root mass in all three species examined here by 40–59% by the end-of-drought harvest. For N, drought significantly decreased only the concentration of the main nitrate-uptake protein, NRT1, in big bluestem (*ca*. 60%), although there were non-significant decreases in the concentration of the main ammonium-uptake protein, AMT1, in barley (18%) and big bluestem (17%). In addition to the concentration of N- and P-uptake proteins per g root, the relative P-uptake rate per PHT1 protein (i.e., activity per protein) was reduced by drought in all three species. Similar calculations for NRT1 or AMT1 were not possible, as explained in the methods; however, since drought had only small effects on the concentration on N-uptake proteins, but had large negative effects on N-uptake rate, then overall, drought must have decreased the activity of N-uptake proteins. The reason for the observed decreases in nutrient-uptake protein activity is not known, but could include damage to nutrient-uptake proteins or spatial mismatches within root tissue between regions of continued water uptake vs. nutrient availability during drought. Previous studies have demonstrated that drought can impact the expression of nutrient-uptake-protein genes (discussed below), but we are not aware of any other studies that have measured protein levels of nutrient-uptake proteins or their activity in response to drought [15,30,32].

Drought-related decreases in the levels of N- and P-uptake proteins per gram root occurred despite increases in levels per unit total root protein, because of larger decreases in the concentration of total protein per gram root. The upregulation of NRT1, AMT1, and PHT1 proteins per unit total root protein during drought is consistent with a recent study observing increases in mRNA levels of NRT1.2, AMT1.1, and AMT1.3 during drought in corn (NRT1.1, which was also measured, did not increase) [32]. In contrast, other studies have observed no effects (sorghum) or even negative effects of drought on mRNA levels of nutrient-uptake proteins (Zn transporter, corn; Si transporter, rice; NO_3_ and SO_4_ transporters, grape) [30]. However, given that nutrient-uptake proteins can be post-transciptionally regulated [28,29], it is important to measure protein levels of nutrient-uptake proteins, in addition to mRNA levels, to understand how drought affects expression of nutrient-uptake proteins and nutrient-uptake rate.

Previous studies have demonstrated that drought can decrease the protein concentration of plant tissues [33,34,35], as observed in this study. Drought-related decreases in plant protein concentration can occur for several reasons, including: (1) decreases in N assimilation [15,30]; (2) decreases in protein synthesis [13]; and (3) increases in protein degradation, such as due to oxidative damage, which occur during drought [36]. We hypothesize that the maintenance of root protein concentration during drought would have functional benefits for plants, such as by increasing nutrient-uptake-protein levels and nutrient-uptake rates, as well as nutritional benefits for herbivores, by improving food quality.

In this study, in general, similar effects of drought were observed in all three species: drought-sensitive barley and corn, and drought-tolerant big bluestem. Each species was subjected to a roughly-comparable relative degree of drought stress, as evidenced by similar reductions in biomass by the end-of-drought harvest (44, 28, and 38% for barley, corn, and big bluestem, respectively), though, of course, similar drought effects were observed at lower leaf water potentials in drought-tolerant big bluestem (−2.51 MPa) than in drought-sensitive barley (−1.68 MPa) and corn (−1.91), as is typical [26,35,37]. Consequently, drought reduced total plant %N and %P to a similar extent in the three species. Yet while effects of drought on nutrient relations were generally similar, there were some notable differences among drought-sensitive and -tolerant species. For example, drought-related decreases in N- and P-uptake rates were greater for barley than for corn and big bluestem. In fact, N-uptake rates for barley were negative, indicating that barley plants lost more N than they took up from the soil over the course of the drought, likely via increased fine-root turnover [37] or volatilization [38]. In contrast, drought-related decreases in the concentration of total protein and the main P-uptake protein, PHT1, per gram root were largest in drought-tolerant big bluestem, intermediate in corn, and smallest in drought-sensitive barley. Big bluestem was also the only species to exhibit a decrease in NRT1 per gram root. However, larger decreases with drought in PHT1 and NRT1 per gram root were likely offset by larger increases in root-to-shoot mass in big bluestem, compared to corn (intermediate increases) and barley (lowest increases). As is already well known, plant nutrient uptake is dependent on both root quantity and root quality (uptake per g or cm^2^), and the relative importance of quantity vs. quality varies among nutrients [26,30].

In summary, our results indicate that (1) decreases in plant nutrient concentration with drought are likely caused, at least in part, by decreases in the concentration (P only) or activity (P and N) of nutrient-uptake proteins in roots; (2) decreases in the concentration of nutrient-uptake proteins per g root can be driven by decreases in the concentration of total root protein; and (3) these effects of drought occur in both drought-sensitive and -tolerant species, and they often become more severe as drought stress progresses. With climate change, the frequency, intensity, and duration of drought will often increase. We hypothesize that efforts to improve the tolerance of crops to climate change might include adaptations that help roots maintain bulk protein levels or increase their expression of nutrient-uptake proteins, which will minimize decreases in root nutrient uptake and tissue nutrient concentration with drought. However, future studies that investigate the effect of upregulated expression of nutrient-uptake protein concentration on nutrient-uptake rate and tissue nutrient concentration will be required to confirm the role of root nutrient-uptake protein concentration to maintenance of plant nutrient status during drought.

## 4. Materials and Methods

### 4.1. Plant Material, Growth Conditions, and Harvesting

This research was conducted using the grass family as a model system, specifically *Hordeum vulgare* (barley), a drought-sensitive C_3_ grass, *Zea mays* (maize or corn), a drought-sensitive warm-season C_4_ grass, and *Andropogon gerardii* (big bluestem), a drought-tolerant warm-season C_4_ grass. Seeds were sown in calcined clay in drainable trays in the greenhouse, and the trays were watered daily. After plants reached the 4-to-5-leaf stage, seedlings were transplanted into large pots (10-cm diameter × 50-cm depth, with mesh bottoms) containing calcined clay, and each pot was placed in its own shallow tray (15 × 15 × 3 cm). Following transplantation, plants were watered as needed and, after three days, fertilized with a complete nutrient solution [Hoagland’s: 6.2 mM KNO_3_, 1 mM KH_2_PO_4_, 1 mM K_2_HPO_4_, 2 mM CaCl_2_, 2 mM MgSO_4_, 71 µM Fe-DTPA (diethylene-triamine-penta-acetic acid), 10 µM MnCl_2_, 50 µM H_3_BO_3_, 6 µM CuSO_4_, 6 µM ZnSO_4_, 1 µM Na_2_MoO_4_; pH = 6.0; full-strength for corn and barley, and 1/2-strength for big bluestem, as it is adapted to low-nutrient conditions]. Temperature in the greenhouse was maintained at ca. 30/23 ± 2 °C (day/night), and light levels ranged from 200–1800 µmol m^−2^ s^−1^ photosynthetically active radiation (PAR), depending on cloud cover, with a 15-h photoperiod. If the light level went below 300 PAR, the day-extension lighting automatically went on [i.e., at <15% of full sunlight, provided by 250-W high-pressure sodium (GE Lucalox) and 400-W metal-halide (Sylvania Metalarc) lamps].

Drought was imposed in the greenhouse by withholding water, and the rate and severity of drought stress were controlled by daily monitoring of plant + soil + pot mass and stomatal conductance, and then partially replacing the water lost the previous day. This method to impose drought, as in [37], is intended to expose plants to a gradually-increasing drought stress of duration similar to that in a field situation, wherein plants have the opportunity for acclimation. Based on preliminary trials, 100 mL of ¼-strength Hoagland solution was given every other day to the plants. Immediately prior to initiating the drought treatment, six random plants were harvested as “time-zero” control plants for determination of nutrient-uptake rates during the experiment. Plants were harvested at 14 or 18 days (for barley and corn/big bluestem, respectively) after the start of drought (at 94, 26, and 37% stomatal closure for barley, corn, and big bluestem, respectively), and after 30 days of drought (for corn and big bluestem; stomatal closure = 80% in corn and 71% in big bluestem) (Figure S1). Because barley was so drought-sensitive relative to the other two species, we were only able to collect data for the end-of-drought harvest for barley. Plant water status (Ψ_leaf_, measured as xylem pressure potential) was measured with a pressure chamber. Leaf water potential (MPa, mean + 1SE, control vs. drought) at the end of drought was: barley (−0.34 + 0.02 vs. −1.68 + 0.03), corn (−0.84 + 0.07 vs. −1.91 + 0.04), and big bluestem (−1.23 + 0.03 vs. −2.51 + 0.05). Plants were rotated every three days to avoid position effects. Each species was grown and studied at different times, and, hence, was an independent experiment. At each drought harvest (mid and end of drought), a subset of well-watered control and drought-stressed plants were harvested (n = 5–7 per treatment). The extent of drought stress was assessed by daily measurements of leaf stomatal conductance to water vapor (G_s_) and net photosynthesis (P_n_; net CO_2_ assimilation), and by determining leaf water status prior to harvest. Measurements of G_s_ and P_n_ were made on 3–5 randomly selected plants at the same time each day (12 p.m.–1 p.m.), using a portable photosynthesis system with infrared gas analyzer, equipped with a 6-cm^2^ leaf chamber and CO_2_, light, and temperature control (Model 6400, LiCOR, Lincoln, NE, USA); measurements were made within 1 min of enclosing leaves within the cuvette and after reaching stable rates of gas-exchange. For P_n_, recently-expanded, illuminated attached leaves were measured while maintaining CO_2_ at 400 ppm and light intensity at 1000 µmol m^−2^ s^−1^ PAR.

At harvest, plants parts were separated into shoots and roots (roots after washing with deionized water). The root system was divided longitudinally into two halves, and one half was weighed (to determine fresh mass) and then oven-dried for nutrient analysis, while the other half was used for protein analysis. Dry biomass of shoots and roots was determined after oven drying at 65 °C for at least 72 h. Fresh root tissue for protein analysis was immediately frozen in liquid N_2_ after harvest and stored at −80 °C; dry mass of this tissue was estimated from fresh-to-dry mass ratio of the other half of the root system.

### 4.2. Nutrient and Protein Analysis

The C and N concentrations (% dry mass) of homogenized shoot and root tissue were determined using the combustion mass-spectrometry (MS) technique, while other micro and macronutrients were measured using the inductively-coupled-plasma optical-emission spectroscopy (ICP-OES) technique, as explained in [39]. Nutrient concentrations were then used to determined total nutrient amounts in the plants (total amount in plant = tissue concentration × tissue mass, for shoot + root). Nutrient-uptake rates per g root dry mass were determined from the increase in total plant nutrient content from one harvest to the next, divided by g root dry mass at the later harvest, divided by days between harvests. For example, N or P uptake rate per g dry root on day-18 harvest was determined by the following equation: N or P uptake rate (mg g^−1^ dry root day^−1^) = (total plant N or P content on day 18–total plant N or P content on day 0)/root dry mass on day 18/18 days (for the end-of-drought harvest, data for days 25 and 18 were used).

For protein analysis, total root protein was extracted according to [40] by grinding 0.5 to 1 g of fresh tissue in liquid N_2_ in a mortar and pestle and then in 2 to 4 mL of extraction buffer [0.2 M Tris pH 8, 1% SDS (sodium dodecyl sulphate), 0.7 M sucrose, 5 mM EDTA (ethylene diamine tetra-acetic acid), 2% β-mercaptoethanol (BME), 1 mM leupeptin, and 1 mM PMSF (phenyl methyl sulfonyl fluoride)]. The homogenate was then transferred to 15-mL tubes and an equal volume of phenol was added, and then the tubes were incubated for 15 min at room temperature on ice, with intermittent vortexing. Then, tubes were centrifuged at 10,000 *g* for 10 min at 4 °C to separate aqueous and organic phases. The upper phenol phase was recovered and washed with an equal volume of extraction buffer and again centrifuged as above. The supernatant was then stored overnight at −20 °C in 5 volumes of chilled 0.1 M ammonium acetate. After overnight incubation at −20 °C, root proteins were pelleted by centrifugation. The resulting pellets were washed twice with ammonium acetate, then with 80% acetone, and followed by final two washes with 100% acetone. Finally, the pellets were dried under room temperature and re-solubilized in re-solubilizing buffer (62.5 mM Tris pH 6.8, 0.5% SDS, and 20% glycerol). Total root protein concentration per g of fresh root was determined using a colorimetric assay (DC protein assay, Bio-Rad, Hercules, CA, USA); bovine serum albumin (BSA) was used as the protein standard.

The relative concentrations (per unit total root protein) of specific nutrient-uptake proteins (NRT1, AMT1, and PHT1 proteins) were determined using protein-specific antibodies and enzyme-linked immuno-sorbent assay (ELISA). For ELISA, equal total root protein was loaded per well, background from non-specific binding was subtracted using pre-immune serum, and all samples were relativized to a standard root-protein extract. Primary antibodies were detected colorimetrically, using secondary antibody conjugated to alkaline phosphatase. Using root fresh-to-dry mass ratio, the relative concentration of specific nutrient-uptake proteins per unit total root protein was converted to concentration per g root dry mass. Then, the apparent relative uptake rate per transporter was determined from nutrient uptake rate per g of root and the relative concentration of transporter per g of root (i.e., nutrient uptake rate per g root = concentration of nutrient-uptake protein per g root × rate per transporter). Mean relative uptake rate per transporter was calculated only for PHT1, since it is well-established that PHT1, a phosphate transporter, is the main P-uptake protein in roots, that roots take up P primarily as phosphate [29], and P was provided only as phosphate in this study. Since the plants in this study were provided N in both nitrate and ammonium form, and the amount or proportion of N taken up as nitrate vs. ammonium is unknown, we cannot estimate the relative uptake rate per unit protein for NRT1 vs. AMT1.

Rabbit polyclonal antisera were generated using oligopeptide antigens of conserved domains of each nutrient-uptake protein. Protein sequences of NRT1, AMT1, and PHT1 families were collected using open-access databases (e.g., NCBI, TAIR), aligned with CLUSTAL W, and further analyzed for the conserved domains across various plant species using DNASTAR software (MegAlign). Potential hydrophobicity of the selected peptide sequences of each N-uptake protein was analyzed with the ProSite database (http://prosite.expasy.org). The oligopeptide antigens were: NRT1-TGGLKSSVSGFGSDQFDESD; AMT1-KLLRISAEDEMAGMDLTRH; PHT1-GDYPLSATIMSEYANKKTRG. These peptide sequences are highly-conserved across species, including in the model monocots corn/rice/barley, and within a species they are found in most isoforms of each uptake-protein type, especially the most-abundant and important isoforms in roots (i.e., NRT1.1 and NRT1.2, AMT1.1 and AMT1.2, and PHT1.1–1.4) [28,29,41,42]. The specificity of antisera was confirmed by western-blotting, comparing results for pre-immune vs. immune sera, antigen-purified vs. crude sera, and against tomato and *Arabidopsis thaliana* samples.

### 4.3. Statistical Analysis

Since each species was investigated in its own separate independent experiment, we analyzed data from each species individually (n = 5–7), using analysis-of variance (ANOVA), with harvest times and watering treatments as fixed factors. For corn and big bluestem, we used two-way ANOVA [two harvest times (mid, late) × two watering treatments (control, drought)]; for barley, we used one-way ANOVA (two watering treatments). Tukey’s post-hoc test was used for multiple comparisons, if ANOVA results for drought × harvest were significant. Results were considered significant if *p* < 0.10. Analyses were conducted using JMP 12 software (SAS Institute Inc., Cary, NC, USA).

## Supplementary Materials

The following are available online at http://www.mdpi.com/2223-7747/7/2/28/s1, Figure S1: Effects of control vs. drought treatments on leaf stomatal conductance to water vapor (G_s_) in barley, corn, and big bluestem (BBS). Each bar represents mean + 1 SE (standard error). Within each panel, bars not sharing the same letters are significantly different. For each response variable, significant effects of drought (ANOVA), across both harvest in corn and big bluestem, are indicated with an asterisk (*). Table S1: Results (*p* values) from ANOVA statistical analyses for Barley. Table S2: Results (*p* values) from ANOVA statistical analyses for Corn. Table S3: Results (*p* values) from ANOVA statistical analyses for Big Bluestem.
