# Supplementary material for: Effects of Drought on Nutrient Uptake and the Levels of Nutrient-Uptake Proteins in Roots of Drought-Sensitive and -Tolerant Grasses
Source: Plants (Basel). 2018 Mar 30;7(2):28. doi: 10.3390/plants7020028 (PMC6027393; doi:10.3390/plants7020028)
Supplement: Supplementary file 1 [file plants-07-00028-s001.pdf]

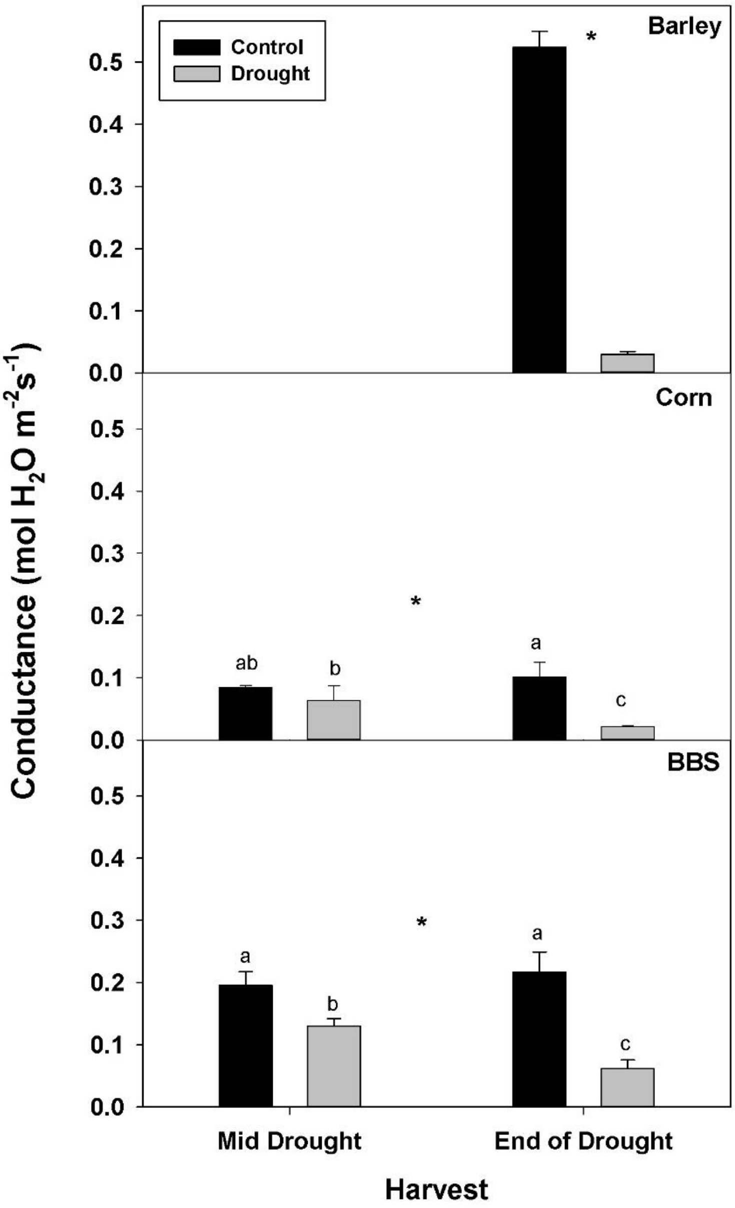

Figure S1. Effects of control vs. drought treatments on leaf stomatal conductance to water vapor (G_s) in barley, corn, and big bluestem (BBS). Each bar represents mean + 1 SE (standard error). Within each panel, bars not sharing the same letters are significantly different. For each response variable, significant effects of drought (ANOVA), across both harvest in corn and big bluestem, are indicated with an asterisk (*).

Table S1. Results (*p* values) from ANOVA statistical analyses for Barley.

Response Variable	Drought Effect
Stomatal conductance	0.0001
Shoot dry mass	<0.0001
Root dry mass	0.0164
Total dry mass	0.0004
Shoot:root mass	0.0002
Root %N	0.0003
Shoot %N	0.0002
Total plant %N	<0.0001
Root % P	0.0001
Shoot % P	0.0017
Total plant %P	0.0005
N uptake rate	<0.0001
P uptake rate	0.0375
Total root protein	0.0001
NRT1 per unit total protein	0.0005
NRT1 per g of dry root	0.7789
AMT1 per unit total protein	0.0944
AMT1 per g of dry root	0.2217
PHT1 per unit total protein	0.0538
PHT1 per g of dry root	0.0013
PHT 1 Activity	0.0001

Table S2. Results (*p* values) from ANOVA statistical analyses for Corn.

Response Variable	Harvest (H) Effect	Drought (D) Effect	H × D Effect
Stomatal conductance	0.1983	<0.0001	0.0049
Shoot dry mass	<0.0001	0.2412	0.0001
Root dry mass	<0.0001	0.7564	0.8696
Total dry mass	<0.0001	0.4547	0.0003
Shoot:root mass	0.0001	0.0806	<0.0001
Root %N	0.3394	0.0483	0.2084
Shoot %N	0.0941	0.0009	0.0003
Total plant %N	0.6270	0.0027	0.0005
Root %P	<0.0001	<0.0001	0.0001
Shoot %P	0.0010	0.0166	0.2693
Total plant %P	0.0148	0.0010	0.7350
N uptake rate	<0.0001	0.0070	<0.0001
P uptake rate	0.0262	0.0066	0.6972
Total root protein	0.6256	0.0384	0.5413
NRT1 per unit total protein	0.1480	0.0136	0.4886
NRT1 per g of dry root	0.6050	0.4629	0.5316
AMT1 per unit total protein	0.2314	<0.0001	0.0041
AMT1 per g of dry root	0.0036	0.0027	0.0114
PHT1 per unit total protein	0.0134	0.0003	0.0831
PHT1 per g of dry root	0.0004	0.0066	0.0136
PHT 1 Activity	0.0009	0.3917	0.4386

Table S3. Results (*p* values) from ANOVA statistical analyses for Big Bluestem.

Response Variable	Harvest (H) Effect	Drought (D) Effect	H × D Effect
Stomatal conductance	0.0153	<0.0001	0.0001
Shoot dry mass	<0.0001	0.0656	0.0002
Root dry mass	0.0160	0.5275	0.8050
Total dry mass	0.0002	0.2744	0.0871
Shoot:root mass	0.0309	0.2334	0.0196
Root %N	0.4172	0.1488	0.3299
Shoot %N	0.6691	0.0098	0.5953
Total plant %N	0.6270	0.0027	0.0005
Root % P	0.9583	0.0214	0.4946
Shoot % P	0.4103	0.1229	0.3896
Total plant %P	0.5910	0.0330	0.8095
N uptake rate	0.6144	0.0023	0.2154
P uptake rate	<0.0001	0.2556	0.0079
Total root protein	0.4554	0.6535	0.0457
NRT1 per unit total protein	0.5385	<0.0001	<0.0001
NRT1 per g of dry root	0.0029	0.0674	0.0002
AMT1 per unit total protein	0.6239	0.0004	0.1297
AMT1 per g of dry root	0.1011	0.0032	0.0038
PHT1 per unit total protein	0.0069	0.4307	0.8007
PHT1 per g of dry root	0.0227	0.2245	0.1603
PHT 1 Activity	<0.0001	0.0640	0.4134
